# Recombinant Prion Protein Refolded with Lipid and RNA Has the Biochemical Hallmarks of a Prion but Lacks In Vivo Infectivity

**DOI:** 10.1371/journal.pone.0071081

**Published:** 2013-07-30

**Authors:** Andrew G. Timmes, Roger A. Moore, Elizabeth R. Fischer, Suzette A. Priola

**Affiliations:** 1 Laboratory of Persistent Viral Diseases, Rocky Mountain Laboratories, National Institute of Allergy and Infectious Disease, National Institutes of Health, Hamilton, Montana, United States of America; 2 Electron Microscopy Unit, Research Technologies Branch, Rocky Mountain Laboratories, National Institute of Allergy and Infectious Disease, National Institutes of Health, Hamilton, Montana, United States of America; Dulbecco Telethon Institute and Mario Negri Institute for Pharmacological Research, Italy

## Abstract

During prion infection, the normal, protease-sensitive conformation of prion protein (PrP^C^) is converted via seeded polymerization to an abnormal, infectious conformation with greatly increased protease-resistance (PrP^Sc^). In vitro, protein misfolding cyclic amplification (PMCA) uses PrP^Sc^ in prion-infected brain homogenates as an initiating seed to convert PrP^C^ and trigger the self-propagation of PrP^Sc^ over many cycles of amplification. While PMCA reactions produce high levels of protease-resistant PrP, the infectious titer is often lower than that of brain-derived PrP^Sc^. More recently, PMCA techniques using bacterially derived recombinant PrP (rPrP) in the presence of lipid and RNA but in the absence of any starting PrP^Sc^ seed have been used to generate infectious prions that cause disease in wild-type mice with relatively short incubation times. These data suggest that lipid and/or RNA act as cofactors to facilitate the de novo formation of high levels of prion infectivity. Using rPrP purified by two different techniques, we generated a self-propagating protease-resistant rPrP molecule that, regardless of the amount of RNA and lipid used, had a molecular mass, protease resistance and insolubility similar to that of PrP^Sc^. However, we were unable to detect prion infectivity in any of our reactions using either cell-culture or animal bioassays. These results demonstrate that the ability to self-propagate into a protease-resistant insoluble conformer is not unique to infectious PrP molecules. They suggest that the presence of RNA and lipid cofactors may facilitate the spontaneous refolding of PrP into an infectious form while also allowing the de novo formation of self-propagating, but non-infectious, rPrP-res.

## Introduction

The fatal, transmissible neurological diseases scrapie in sheep, Creutzfeldt-Jakob Disease in humans and bovine spongiform encephalopathy in cattle are known as transmissible spongiform encephalopathies (TSEs). They are also known as prion diseases because they are associated with abnormal proteins known as prions that are derived from the mammalian prion protein (PrP). During prion infection, the normal protease-sensitive and soluble host prion protein (PrP^C^) is converted to an infectious, protease-resistant and insoluble form (PrP^Sc^) which has a different tertiary structure. PrP^Sc^ can accumulate over time in tissues of the central nervous system and immune system and, once it reaches pathogenic levels in the brain, trigger a prion disease. PrP^Sc^ accumulation is dependent upon the ability of PrP^Sc^ to replicate by inducing the conformational conversion of PrP^C^ to more PrP^Sc^, a process known as seeded polymerization (for review see [1]). Detergent insolubility, increased resistance to degradation by proteases, an increased amount of beta-sheet structure relative to PrP^C^, and especially the ability to self-propagate are all considered hallmarks of infectious prions. However, the mechanisms which underlie the refolding of PrP^C^ into PrP^Sc^ remain poorly understood.

Over the last several years, multiple model systems have been developed which are capable of producing infectious PrP in vitro. Techniques such as Protein Misfolding Cyclic Amplification (PMCA), which allows for the self-propagation of PrP^Sc^ using uninfected brain homogenate [2] or purified brain-derived PrP^C^ [3] as PrP^C^ substrates as well as other purified or synthetic cofactors [3], have been useful in studying the molecular basis of prion species barriers [4], [5] and strains [5]. The amount of prion infectivity generated is often lower than that found associated with brain-derived PrP^Sc^ [3], [6], [7] although infectivity levels similar to those in brain have been reported in PMCA reactions for some prion strains [8] and may be dependent upon the size of the PMCA product [9]. The relatively low yield and complexity of PMCA reactions containing brain homogenate, as well as the difficulties associated with obtaining structural information using glycosylated PrP^Sc^ [10], make detailed studies of the refolding mechanism leading to infectious prions difficult.

One of the best ways to elucidate the mechanisms of prion formation would be to generate infectious prions *in vitro* spontaneously, i.e. in the absence of a starting seed of PrP^Sc^, using highly purified *E.coli*-derived recombinant PrP (rPrP). In recent years, multiple examples of prion infectivity spontaneously generated *in vitro* using rPrP have been reported [11]–[17]. However, in most of these studies, the amount of infectivity generated appears to be extremely low. For example, recombinant hamster PrP exposed to heat and bovine serum albumin [12] or used as a substrate in PMCA [13] yielded low levels of hamster prion infectivity. On first passage, amyloid fibrils of recombinant mouse or hamster PrP formed spontaneously can induce clinical disease in transgenic mice overexpressing mutant PrP^C^ [11], or trigger PrP^Sc^ formation in either hamsters [15] or transgenic mice expressing both wild-type and anchorless forms of mouse PrP^C^ [16]. Depending upon the sample being tested, in these latter studies clinical disease is observed with a high attack rate only upon second [15], [16] or third [16] passage. Other experiments have shown that spontaneously formed recombinant mouse PrP takes from 470–700 days to induce disease following inoculation into transgenic mice overexpressing wild-type PrP^C^ [17]. The fact that reactions using rPrP appear to generate very little infectivity suggests that they also would be difficult to use to study the mechanisms of PrP^Sc^ formation or the structure of infectious prions.

RNA can stimulate the conversion of PrP^C^ into PrP^Sc^ in the absence of a starting PrP^Sc^ seed [18] and can facilitate the spontaneous conversion of PrP^C^ into PrP^Sc^ which can then transmit disease to hamsters [3]. Recently, it has been shown that unseeded PMCA reactions combining mouse rPrP, mouse liver RNA and lipid [14] or seeded PMCA reactions containing mouse rPrP, synthetic RNA and lipid [19] yield a form of protease-resistant rPrP (rPrP-res) that is able to induce clinical disease in wild-type mice. The average incubation time of the disease induced by rPrP-res in these studies was approximately 130 and 200 days, respectively, suggesting that the reactions contained significant prion infectivity. When seeded with rPrP-res, PMCA reactions containing rPrP and phospholipid, but no RNA, also produce infectious rPrP-res capable of infecting wild-type mice but only after an incubation time of almost 400 days [20]. Taken together, these data suggest that RNA and lipid are important cofactors in facilitating the spontaneous conversion of rPrP into a highly infectious form.

In the present study, we have used unseeded PMCA reactions containing recombinant mouse PrP, mouse liver RNA, and lipid to make rPrP-res. The rPrP-res generated was insoluble with an N-terminal truncation similar to that of brain-derived PrP^Sc^. However, it was unable to induce either sub-clinical or clinical disease in long-term animal bioassays and was incapable of inducing the formation of PrP^Sc^ in cells susceptible to prion infection. Furthermore, neither variations in cofactor concentration nor the use of two different preparations of rPrP yielded rPrP-res capable of inducing PrP^Sc^ formation in cells. Our data are consistent with the hypothesis that PrP is able to assume multiple protease-resistant structures and suggest that the ability to self-propagate into a protease-resistant insoluble conformer is common to both infectious and non-infectious forms of PrP. Furthermore, the fact that RNA and lipid cofactors do not necessarily induce PrP to spontaneously refold into an infectious form suggests that the de novo generation of infectious PrP may result from a stochastic event which is only made more likely by the addition of RNA and lipid.

## Materials and Methods

### Ethics Statement

All animal experimental protocols were reviewed and approved by the Rocky Mountain Laboratories Animal Care and Use Committee (Protocol #2010-64). The Rocky Mountain Laboratories are fully accredited by the American Association for Laboratory Animal Care and this study was carried out in strict accordance with the recommendations in the Guide for the Care and Use of Laboratory Animals of the National Institutes of Health.

### HPLC-Mass Spectrometry for Intact Protein

Proteins were injected into a reverse phase HPLC (Agilent 1100 series HPLC, Agilent Technologies) with a Zorbax 300SB-C18 (2.1×50 mm, 3.5 µM, Agilent Technologies) and introduced into the mass spectrometer as described [21], [22]. Positive ion Electrospray Ionization (ESI) mass spectra for intact protein were obtained with an Agilent 6224 mass spectrometer equipped with an ESI interface and a time-of-flight (TOF) mass detector (Agilent Technologies). Mass spectra were analyzed and deconvoluted as described [23], using an Agilent software MassHunter version B.04.00 (Agilent Technologies).

### Recombinant PrP Purification

The cloning and expression of recombinant wild-type mouse PrP has been described previously [16]. Two methods were used to purify recombinant PrP (rPrP). The first method was a modified form [24] of the protocol by Zahn et al. [25]. Briefly, approximately 2 g of bacterial cell pellets equivalent to 250 ml of LB-Miller growth medium were lysed with BugBuster using lysonase (EMD Biosciences). Inclusion bodies were isolated by centrifugation, washed, and then solubilized in denaturing buffer (6 M guanidine, 100 mM sodium phosphate, 10 mM Tris, pH 8.0) without reducing agents. The denatured rPrP was again subjected to centrifugation and then mixed with 15 g of NiNTA resin (Qiagen) prior to on-column refolding using a linear gradient into refolding buffer (100 mM sodium phosphate,10 mM Tris, pH 8.0). Refolded rPrP was further washed in refolding buffer to remove any residual guanidine prior to elution with 500 mM imidazole, 100 mM sodium phosphate, 10 mM Tris pH 5.8. The eluted rPrP was filtered, dialyzed against 10 mM ammonium acetate, pH 4.5, and stored at −80°C. Prior to use, rPrP was thawed and dialyzed into water. The mass of rPrP was verified by electrospray intact mass analysis (ESI-MS) to be 23,061.83 Da (Expected: 23,063.3 Da; Figure S1A and C).

A second purification also based on the protocol of Zahn et al. [25], was done in closer accordance to that described by Wang et al. [14], [26]. This purification differed from the first primarily in the use of beta-mercaptoethanol (BME) during PrP refolding, the addition of a cation exchange step, and no dialysis of the purified PrP into 10 mM ammonium acetate, pH 4.5. Briefly, 10 mM BME was added to the denaturing buffer prior to the on-column refolding procedure described above. After washing and elution with 500 mM imidazole, fractions containing rPrP were dialyzed first against 10 mM sodium phosphate, pH 5.8 and then against water. A subsequent cation exchange chromatography (CM Sepharose) step was performed in which rPrP was eluted using a linear gradient from 0 to 500 mM sodium chloride in 10 mM sodium phosphate, pH 5.8 buffer. The purified protein was dialyzed against water and stored at −80°C until needed. Recombinant PrP purified using this second protocol will be referred to as rPrP2 throughout the manuscript. The mass of rPrP2 was verified by ESI-MS to be 23,061.85 Da (Expected: 23,063.3 Da; Figure S1B and D).

For both rPrP and rPrP2, purity was estimated to be at least 99% by SDS-PAGE and silver stain. Approximately 99% of the protein was monomeric with an intact disulfide bond and no truncated forms were detected (Figure S1G). Smaller peaks were present in both preparations that may represent oxidation and/or sodium and iron adducts (Figure S1E and F). However, both iron adducts [27] and oxidation [28], [29] are known artifacts of ESI-MS and it is not clear whether these modifications were present in our rPrP samples prior to analysis.

### PMCA

The components of the PMCA reaction were combined according to Wang, et al., 2010 [14]. Purified rPrP was centrifuged for 1 hr at 100,000×*g* and the supernatant containing soluble rPrP was removed. Recombinant PrP was added to a final concentration of 5 µg/ml to the synthetic lipid 1-palmitoyl-2oleoyl-sn-glycero-3-phospho-(1′-sn-glycerol) (sodium salt) (POPG, 4.4 µg/ml final concentration). After a 10 minute incubation, the rPrP/POPG mixture was diluted into Tris/NaCl/Triton X-100 to yield a solution with 0.28% Triton X-100, 10 mM Tris-HCl, and 150 mM NaCl. After a 5 minute incubation, mouse liver extract containing RNA was added to a final concentration of 30 µg/ml and the solution thoroughly mixed. Without further incubation, the completed reaction mixture was aliquoted and frozen at −80°C for use in future experiments.

For PMCA reactions, the reaction mixture was thawed and subjected over a 24 hr period to 48 cycles of 30 second sonication at 37°C followed by a 30 minute incubation without sonication (one round). The product of the first round of PMCA was used to seed the second 24 hr PMCA round at a dilution of 1∶10 and the process repeated for twenty rounds (i.e. 20 days). At each round, reaction products were digested using proteinase K (PK) as in [14] and analyzed for recombinant, protease-resistant PrP (rPrP-res) by western blotting using the anti-PrP mouse monoclonal antibody 6D11.

### Immunoblots

Samples were loaded onto either 14% NuPAGE gels or 15% Tris-HCl Criterion gels (BIO-RAD). The mouse monoclonal anti-PrP antibodies 6D11 (Covance), POM1 (Prionatus), and 8B4 (Santa Cruz Biotechnology) were used at concentrations of 0.4, 0.5, and 0.2 µg/mL, respectively and diluted in Enhanced Chemiluminescence (ECL) Advance Blocker/Diluent (GE Healthcare) in 10 mM Tris pH 8.0, 150 mM NaCl, 0.05% Tween-20. The secondary antibody sheep anti-mouse IgG conjugated with horseradish peroxidase (GE Healthcare) was used at a dilution of 1∶100,000 for 6D11, 1∶40,000 for POM1, and 1∶10,000 for 8B4 in the same diluent as the primary antibody. Detection was with ECL Advance (GE Healthcare) according to the manufacturer’s instructions. The rabbit polyclonal anti-PrP antibody R20 [30] was used at a 1∶5000 dilution in conjunction with a donkey anti-rabbit IgG secondary antibody conjugated to horseradish peroxidase (GE Healthcare) diluted at 1∶20,000. All blots using R20 were developed using ECL Prime (GE Healthcare).

The detection limit of our immunoblot procedure for PrP was determined by comparison to different dilutions of PrP loaded onto the same gel as the samples being analyzed. Depending upon the experiment, the PrP used for the dilution series was either recombinant PrP, PrP^Sc^ derived from the brain or spleen of a mouse positive for clinical scrapie, or PrP^C^ derived from tissue culture cells. In general, the limit of detection of our immunoblot procedure was sufficient to detect PrP at a level equivalent to 1% of the PrP^C^ in the cell, 1% of the PrP^Sc^ in the brain of a clinically ill mouse, or 10% of the PrP^Sc^ in the spleen of a mouse with clinical scrapie.

### Immunohistochemistry

Sagittal mouse brain sections were fixed and stained as described previously [7] except that the sections were incubated for 2 hours in the human monoclonal D13 anti-PrP antibody and biotinylated anti-human IgG antibody was used as the secondary antibody.

### Transmission Electron Microscopy (TEM)

Samples of the rPrP-res PMCA product inoculated into mice were digested with 100 U/mL Benzonase (Novagen) for one hour at 37°C and then incubated with 10 µg/mL PK (Roche) for 30 minutes at 37°C. Pefabloc (Roche) was added to a final concentration of 4 mM, and the samples were chilled on ice for 5 minutes. Digested samples were transferred to polycarbonate TLA100.3 tubes and spun at 100,000 rpm for one hour at 4°C. The supernatants were decanted and discarded and the pellets resuspended in 167 µL deionized water by cuphorn sonication in a Misonix S3000 sonicator at a power setting of 1.0 for one minute. The resuspended pellets were then centrifuged at the same speed, time, and temperature as above. The second supernatant was also decanted and discarded and the final pellet was resuspended in 30 µL deionized water with sonication. A 5 µL volume of the suspension was applied to freshly glow discharged carbon coated copper grids and allowed to settle for fifteen minutes. Excess fluid was removed by wicking with filter paper and grids were negatively stained by applying 5 µL 1% uranyl acetate in distilled water for one minute, before final wicking. Specimens were viewed at 120 kV on a Tecnai BT Spirit transmission electron microscope (FEI) at a nominal magnification of 49,000×. Digital images were acquired with a Hammamatsu XR-100 side mount digital camera system (Advanced Microscopy Techniques).

Samples of extracted RNA were left untreated or digested for 60 minutes at 37°C either with Benzonase alone (250 U/mL in 0.1×PBS pH 7.2, 1 mM MgCl_2_) or Benzonase plus α-amylase (Sigma, 1∶500 dilution) and amyloglucosidase (Sigma, 1∶500 dilution). The reactions were then filtered through 0.02 µm Anotop filters (Whatman). The final RNA samples, which were 10 times more concentrated than those used in the PMCA reactions, were applied to grids without any ultracentrifugation steps and stained as above.

### Immunoelectron Microscopy

Specimens were applied to freshly glow discharged carbon coated nickel grids as described above. After wicking excess sample, subsequent steps were performed by placing grids specimen-side down on 20 µL droplets on a PELCO® PTFE immunostaining pad. After blocking in 3% bovine serum albumin (BSA), 0.1% Tween-20 in 10 mM Tris pH 8.0 for 10 minutes, specimens were incubated with the anti-PrP mouse monoclonal antibody 6D11 diluted 1∶100 in dilution buffer (1% BSA, 0.1% Tween-20 10 mM Tris/pH 8.0) or with dilution buffer alone for one hour. Grids were washed 3 times for 5 minutes with dilution buffer and incubated for one hour with a sheep anti-mouse antibody conjugated to 10 nm colloidal gold (BBInternational) diluted 1∶25 in dilution buffer. Grids were rinsed two times for 5 minutes each with dilution buffer followed by three 5 minute rinses with distilled water. The grids were then stained with uranyl acetate and imaged as described above.

### Cell Culture

Two cell lines, SN56 derived from septal neurons [31] and CF10+MoPrP, were inoculated with 22L mouse scrapie brain homogenate, rPrP-res or rPrP2-res using the protocol described in Greil et al. [32]. CF10+MoPrP cells are CF10 mouse PrP knockout cells [32] which have been modified to express wild-type mouse PrP [33]. Following inoculation, cells were either analyzed for acute PrP-res formation [34] at passage 0 or passaged at a dilution of 1∶10 into 6-well plates. Up to passage 10, the remaining 9/10 of the cells that had not been passaged were analyzed for PrP^Sc^. Cells were lysed with 200 µL lysis buffer (1 mM Tris-HCl, pH 7.4, 140 mM NaCl, 5 mM EDTA, 0.5% sodium deoxycholate, 0.5% Triton X-100) and the lysate centrifuged at 13,000 rpm for 5 minutes at room temperature. At Passage 10, cells were collected similarly except that all cells from a 75 cm^2^ flask were used. All cell lysate supernatants were stored at −20°C until analyzed.

To assay for PrP^Sc^, cell lysates were thawed and digested with 25 µg/ml PK at 37°C for one hour. Protease digestion was halted by the addition of Pefabloc (Roche) to a final concentration of 2 µM. The protease digested samples were centrifuged at 100,000 rpm for one hour at 4°C. The resultant pellet was resuspended in 20 µL SDS-PAGE sample buffer (65 mM Tris-HCl, pH 6.8, 5% SDS, 3% BME, 10% glycerol, 0.0001% bromophenol blue) and boiled for ten minutes. Unless otherwise noted, for all cell samples the entire sample was loaded into a single lane of an SDS-PAGE gel for analysis by immunoblotting.

### Animal Procedures

Female C57Bl/10 mice or female CD-1 mice from Jackson Laboratories were used for all bioassays. For infection with rPrP-res, a single positive reaction tube was amplified by seeding a new reaction tube with a 1∶10 dilution and then pooling multiple reaction tubes from the same PMCA round. The conversion efficiency of rPrP into rPrP-res for these reactions was 0.3–3% (data not shown). Insoluble rPrP in the PMCA product was concentrated by differential centrifugation as previously described [14], [26]. Samples of inoculum were subjected to PK digestion and the amount of rPrP-res was determined using a standard dilution series derived from a known concentration of rPrP.

All inoculations were performed while the mice were under isoflurane anesthesia. For infection with mouse 22L scrapie, a 10% stock 22L brain homogenate was diluted 1∶10 in PBS/10%FBS and 30 µL inoculated intracerebrally (IC). The IC titer of the 22L stock in C57Bl/10 mice was 2×10^8.8^ infectious units/g of brain. For rPrP-res, both CD-1 mice and C57Bl/10 mice were inoculated IC with 30 µL of undiluted inoculum which contained ∼2 ng of rPrP-res. C57Bl/10 mice were also inoculated IC with serial ten-fold dilutions of rPrP-res diluted into inoculation buffer (10 mM sodium phosphate pH 7.2, 139 mM NaCl, 1 mg/mL bovine serum albumin) and intraperitoneally (IP) with 30 µL or orally (PO) with 100 µL of undiluted rPrP-res. Undiluted and 10^−1^ dilutions of the rPrP-res inoculum were stored at −80°C for 351 days between the inoculation of C57Bl/10 mice and CD-1 mice. Mice were monitored regularly for clinical disease. At either clinical signs of scrapie or at different time points post-inoculation, mice were euthanized by isofluorane overdose and spleens and brains removed for analysis of PrP^Sc^ by immunoblot as previously described [35].

## Results

### Serial Propagation of Recombinant PrP using PMCA

**Figure 1:**
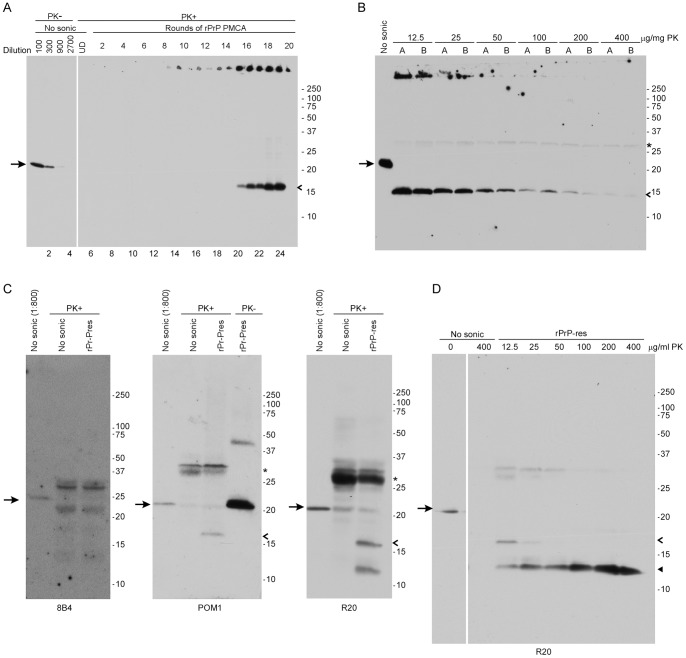
Generation of rPrP-res with a protease-resistant C-terminal core in PMCA reactions containing RNA and lipid. (A) The products of twenty rounds of serial PMCA were PK digested and assayed by immunoblot using the anti-PrP mouse monoclonal antibody 6D11. The number of PMCA rounds is indicated at the top of the gel. Lanes 1–4 represent a standard curve of 1∶100 to 1∶2700 dilutions of the non-PK digested, unsonicated rPrP substrate mixture (No sonic). Based on the standard curve, 0.3–3% of the input rPrP was converted to rPrP-res. Lane numbers are shown at the bottom of the gel. For all panels, the arrow on the left indicates rPrP, the open arrowhead on the right indicates rPrP-res, and molecular mass markers in kDa are indicated on the right. (B) Two independent preparations of rPrP-res (A and B) were digested with increasing concentrations of PK and assayed by immunoblot using the 6D11 antibody. The non-PK digested, unsonicated rPrP substrate mixture (No sonic) was diluted 1∶100 prior to loading. All other samples were loaded without dilution. The asterisk to the right indicates the location of a cross-reactive proteinase K band. (C) rPrP-res was PK-digested and assayed by immunoblot with three anti-PrP antibodies: 8B4 (residues 37–44), POM1 (multiple residues between140–212, see [38]), and R20 (residues 218–231). Unless otherwise indicated, all samples were loaded without dilution. No sonic = non-PK digested, unsonicated rPrP substrate mixture. (D) rPrP-res was digested with varying concentrations of PK and assayed by immunoblot using the anti-PrP antibody R20. The rPrP PK-minus control sample (No sonic) was diluted 1∶100 prior to loading while all PK-digested samples were loaded undiluted. The closed arrowhead indicates the 13 kDa rPrP-res fragment.

Recombinant mouse PrP (rPrP) purified by the method of Atarashi et al. [24] was subjected to PMCA in the presence of POPG lipid and mouse liver RNA as described in the Materials and Methods. Beginning at Round 8 and continuing through Round 20, protease-resistant rPrP (rPrP-res) was detected near the top of the gel (Figure 1A) in 10 out of 40 reaction tubes (25%), suggesting the presence of large SDS and protease-resistant PrP aggregates. The de novo emergence of a protease-resistant fragment of 16 kDa was first visible in the ten positive tubes by round 15 (Figure 1A) and continued to be detectable through round 19. The significantly lower rPrP-res signal in the twentieth round (Figure 1A, lane 25) is an example of the tube to tube variation due to variable delivery of sonication energy that is often seen when using PMCA [36]. By comparing the intensity of the 16 kDa band to a standard dilution series of undigested rPrP (Figure 1A, lanes 1–4), we estimated that approximately 0.3–3% of the total rPrP in the reaction was converted to rPrP-res. Thus, while the reaction conditions used produced rPrP-res capable of self-seeding, the reaction itself was inefficient.

One hallmark of PrP^Sc^ derived from prion-infected animals is its high protease resistance [37]. In order to determine the protease resistance of rPrP-res, two independent samples were digested with concentrations of PK up to 400 µg/mL. The reactions were then analyzed by western blot using the anti-PrP mouse monoclonal antibody 6D11 which recognizes amino acid residues 93–109 in PrP. As shown in Figure 1B, even after digestion with up to 400 µg/mL of PK the 16 kDa rPrP-res was still detectable. Thus, at least some of the 16 kDa rPrP-res generated using the PMCA reaction has a PK resistance similar to that of brain-derived PrP^Sc^ which can be resistant to digestion with 100 µg/ml PK [37].

### The Most Protease-resistant Core of rPrP-res Contains Only the C-terminus of PrP

The amount of 16 kDa rPrP-res product present in the reaction decreased with increasing PK concentrations (Figure 1B). This suggested that either some of the 16 kDa fragment was highly PK-resistant or that digestion with PK was removing the 6D11 antibody epitope at residues 93–109. To determine if other protease-resistant forms of abnormal rPrP were present in the product, immunoblots were also developed with the anti-PrP antibodies 8B4 (N-terminal residues 37–44), POM1 (multiple residues between 140–212, see [38]), and R20 (C-terminal residues 218–231). The 16 kDa rPrP-res fragment reacted with both POM1 and R20 but not with 8B4 (Figure 1C), indicating that it was truncated at the N-terminus and contained PrP sequence from approximately 93–230. This truncation of rPrP-res at the N-terminus by PK is similar to that generated following PK digestion of PrP^Sc^ derived from scrapie-infected brain [39]. By contrast, the high molecular weight PK and SDS-resistant PrP aggregates (Figure 1A and B) did not stain with either POM1 or R20, suggesting that they were not simply aggregates of the 16 kDa PrP protein but rather a PK resistant species missing part of the C-terminus.

The C-terminal antibody R20 detected not only the 16 kDa band but also an approximately 13 kDa rPrP-res fragment not recognized by any of the other antibodies used (Figure 1C). Following digestion with increasing concentrations of PK, the 13 kDa fragment increased in intensity as the 16 kDa fragment decreased in intensity, suggesting that it was derived from the 16 kDa fragment (Figure 1D). The decrease in intensity of the 16 kDa band with increasing PK concentrations (Figure 1B) therefore coincided with the loss of the 6D11 antibody epitope. Thus, our data suggest that rPrP-res has a highly protease-resistant core which does not include the N-terminus but does contain the far C-terminus of PrP.

### rPrP-res is Detergent Insoluble

**Figure 2:**
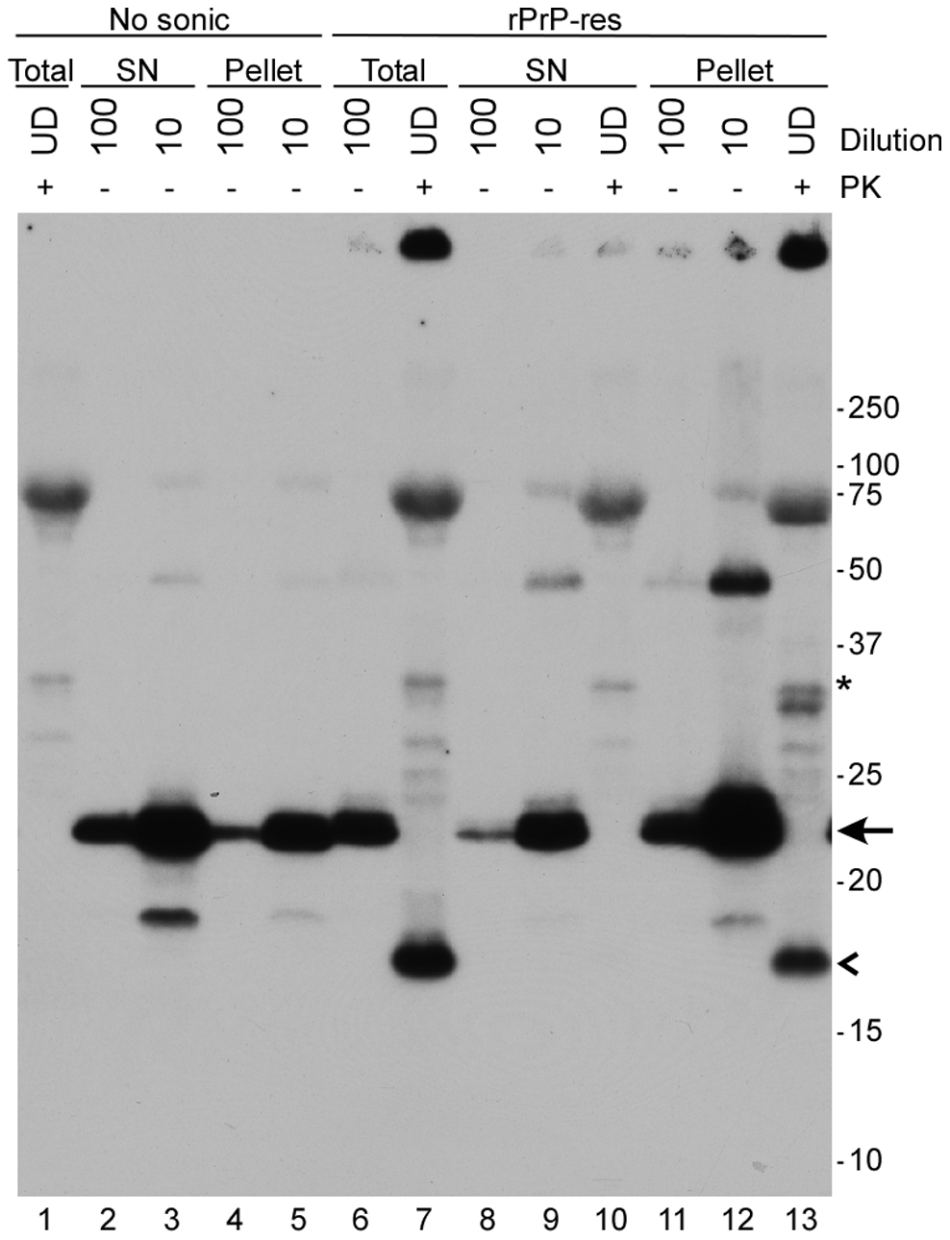
rPrP-res is detergent insoluble. Unsonicated (No sonic) or sonicated (rPrP-res) PMCA substrate mixtures were subjected to ultracentrifugation for one hour at 100,000×*g* and the resulting supernatants (SN) and pellets were assayed by immunoblot with the anti-PrP mouse monoclonal antibody 6D11 either with (+) or without (−) PK digestion. Samples were loaded undiluted (UD) or diluted 1∶10 and 1∶100 as indicated. Molecular mass markers (kDa) are shown on the right. rPrP is indicated by the arrow and the 16 kDa protease-resistant band by an open arrowhead. The band at ∼66 kDa is bovine serum albumin which was added to the samples during methanol precipitation. The asterisk indicates the location of a cross-reactive proteinase K band. Lane numbers are shown at the bottom of the gel. Total = no centrifugation.

Another characteristic feature of disease-associated PrP^Sc^ is detergent insolubility. In order to determine if rPrP-res was also detergent insoluble, PMCA reaction mixtures that had or had not been sonicated were centrifuged at 100,000×*g* for 1 hr at 4°C [40]. While rPrP alone remained soluble and in the supernatant (data not shown), approximately 40% of the rPrP in PMCA reactions that had not been sonicated and thus contained no rPrP-res was found in the pellet (Figure 2, lanes 2–6). By contrast, in PMCA reactions that had been sonicated, rPrP-res was found exclusively in the pellet (Figure 2, compare lanes 10 and 13). Our data show that rPrP experiences a significant loss of solubility due to associations with other components of the substrate mixture and are consistent with a recent report showing that rPrP becomes insoluble following incubation with POPG [20]. However, the fact that all of the rPrP-res generated in the PMCA reaction was found in the pellet suggested that the change in rPrP solubility following sonication was most likely linked to the refolding and oligomerization of rPrP. Thus, similar to PrP^Sc^, rPrP-res is a detergent insoluble aggregate.

### Lack of Detectable Prion Infectivity and PrP^Sc^ in Mice Inoculated with rPrP-res Generated in vitro

Protease-resistant PrP generated in vitro in the absence of PrP^Sc^ can have no infectivity [41] or levels of infectivity that appear to be relatively low [12], [13], [16] or relatively high [14]. In order to assess the infectivity of rPrP-res generated in our PMCA reactions, rPrP-res product from a positive reaction tube was amplified, concentrated by centrifugation, resuspended in PBS/1% BSA, and then inoculated either undiluted or in serial ten-fold dilutions intracerebrally (IC) into C57Bl/10 mice. Undiluted rPrP-res was also inoculated into C57Bl/10 mice intraperitoneally (IP) and orally (PO) and into CD-1 mice IC.

C57Bl/10 mice inoculated IC with 22L mouse scrapie develop clinical scrapie by approximately 150 days post-inoculation (dpi) (Table 1). By contrast, no clinical signs of prion disease were observed up to 626 dpi in C57Bl/10 mice inoculated with rPrP-res by any route (Table 1). Similarly, no clinical signs of prion infection were observed in IC inoculated CD-1 mice up to 367 dpi (Table 1). The lack of clinical disease suggested either that animals inoculated with rPrP-res were subclinically infected or that no infectious rPrP-res was present in the inoculum.

**Table 1:** No evidence of clinical or subclinical infection following inoculation of rPrP-res into mice with different genetic backgrounds.

Inoculum	Mouse strain	Route			Clinical^e^	
			PrP^Sc^			DPI^f^
			Brain	Spleen		
22L^a^	C57Bl/10	IC	+	+	+ (6/6)	154±11
rPrP-res	C57Bl/10^b^	IC	–	–	−(0/8)	>626
rPrP-res	C57Bl/10^c^	IP	–	–	−(0/8)	>626
rPrP-res	C57Bl/10^c^	PO	–	–	−(0/8)	>626
rPrP-res	CD-1^d^	IC	–	–	−(0/8)	>367

a Mice inoculated with a 1∶10 dilution of a 22L mouse scrapie 10% brain homogenate. These mice were not inoculated in the same experiment as the rPrP-res inoculated mice.

b Brains and spleens of two mice tested negative for PrP^Sc^ at 200, 365, and 626 dpi.

c Brains and spleens of two mice tested negative for PrP^Sc^ at 365 dpi.

d Brains and spleens of three mice tested negative for PrP^Sc^ at 367 dpi.

e Numbers in parentheses represent the number of scrapie positive mice over the total number of mice inoculated.

f days post-inoculation (DPI). Numbers represent either average incubation time + SD or days post-inoculation when the experiment was terminated.

**Figure 3:**
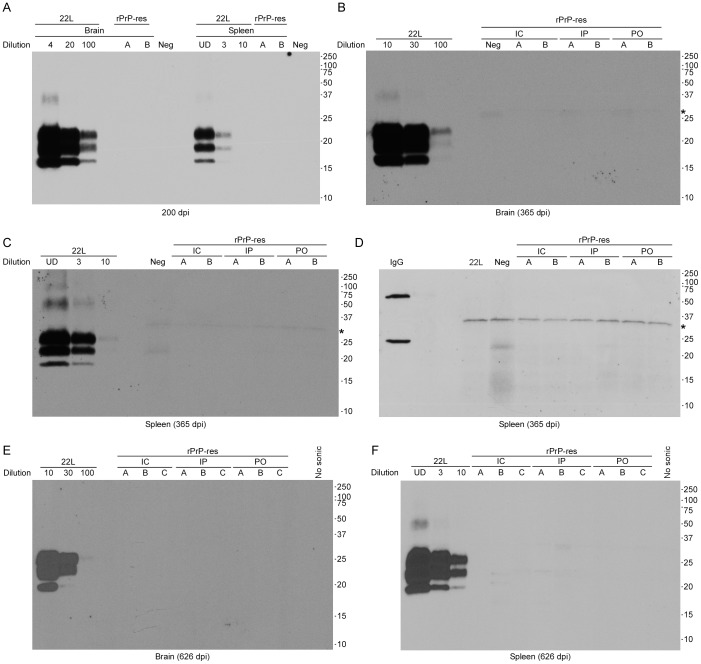
C57Bl/10 mice inoculated with rPrP-res do not accumulate detectable PrP^Sc^ up to 626 days post inoculation. Brain and spleen samples from C57Bl/10 mice inoculated with rPrP-res, 22L mouse scrapie, vehicle alone (Neg), or a mixture of unsonicated rPrP, POPG, and RNA in inoculation buffer (No sonic) were homogenized, PK digested, and assayed by immunoblot using the anti-PrP mouse monoclonal antibody 6D11. (A) Brain and spleen, 200 dpi. (B) Brain, 365 dpi. (C) Spleen, 365 dpi. (D) Spleen, 365 dpi, secondary antibody only. Samples assayed are identical to those in panel C. The first lane shows the reactivity of the secondary antibody with purified mouse IgG heavy and light chains. (E) Brain, 626 dpi. (F) Spleen, 626 dpi. For all panels, tissue samples were loaded undiluted (UD) unless otherwise noted. A standard dilution curve of 22L mouse brain homogenate containing PrP^Sc^ was loaded on each gel as indicated and used to estimate the limit of detection of PrP as detailed in the Materials and Methods. A, B, and C represent individual mice assayed at each timepoint. IC = intracerebral; IP = intraperitoneal; PO = per ora. Molecular mass markers (kDa) are indicated on the right and the asterisk indicates the location of a cross-reactive proteinase K band.

Animals subclinically infected with prions can accumulate PrP^Sc^ in the brain even in the absence of clinical signs [42]. Therefore, we assayed for low levels of PrP^Sc^ in rPrP-res inoculated C57BL/10 and CD-1 mice using immunoblotting. Brains and spleens were collected from rPrP-res inoculated C57Bl/10 mice at 200, 365, and 626 dpi and assayed for PrP^Sc^ by western blot. No PrP^Sc^ was detected in the brain of any mouse inoculated by any route with rPrP-res (Figure 3A, B and E). Brains from CD-1 mice inoculated IC with rPrP-res were also negative at 367 dpi (Figure S2A). In contrast, PrP^Sc^ from the brains of mice clinically ill with 22L mouse scrapie was detectable using 1% of the material used for the rPrP-res brain homogenate samples (Figure 3A, B and E). These results suggest that if rPrP-res inoculated animals had accumulated PrP^Sc^ equivalent to 1% of the amount found in a mouse scrapie-infected brain, it would have been detected using our immunoblot protocol.

PrP^Sc^ can often be detected in the spleens of prion-infected animals before it can be detected in brain [43]. Faint bands were detected in the spleens of rPrP-res inoculated C57Bl/10 mice at 365 and 626 dpi (Figure 3C and F). However, these bands were not PrP-specific but rather the result of cross reactivity of the secondary antibody with IgG (Figure 3D). Similar bands were seen in mice inoculated with control mixtures that had not been subjected to PMCA (Figure 3 and Figure S2B). Spleens from CD-1 mice inoculated IC with rPrP-res were also negative at 367dpi (Figure S2B and S2C). By contrast, PrP^Sc^ from the spleens of mice clinically ill with 22L mouse scrapie was detectable using just 10% of the material used for the rPrP-res spleen samples (Figure 3A, C and F). These results show that if rPrP-res inoculated animals had accumulated PrP^Sc^ in the spleen equivalent to 10% of the amount found in a mouse with clinical scrapie, it would have been detected using our immunoblot protocol.

**Figure 4:**
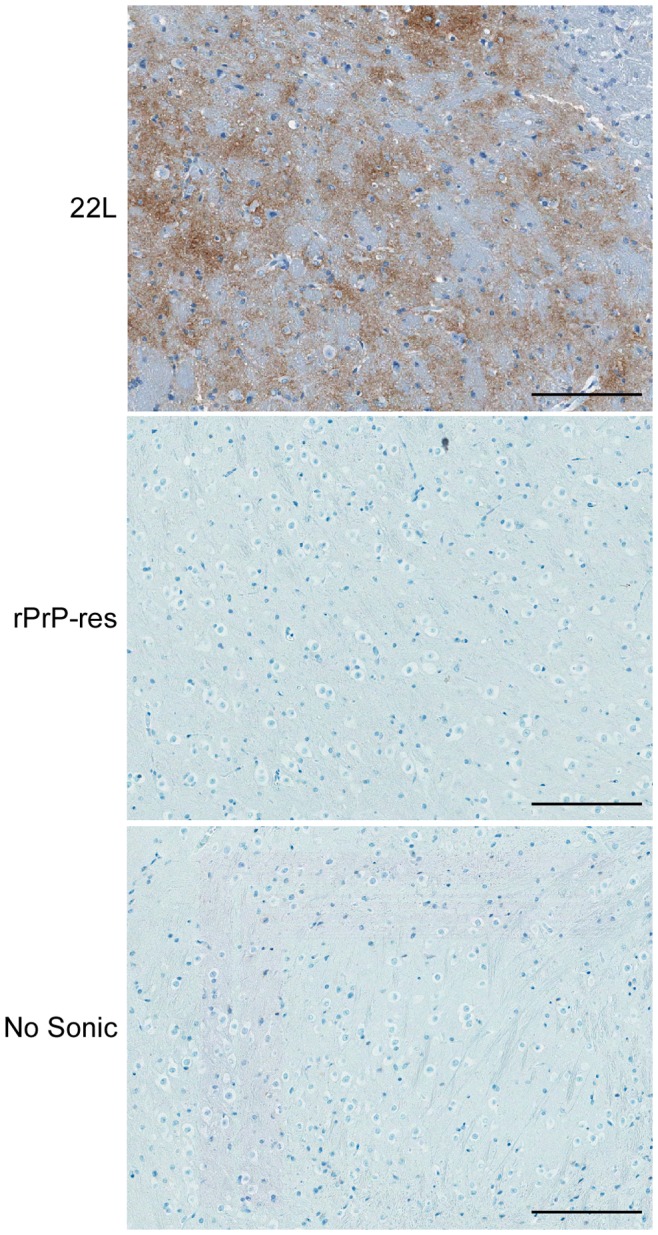
Lack of spongiform change and PrP^Sc^ in C57Bl/10 mice inoculated with rPrP-res. Sagittal sections from C57Bl/10 mice inoculated with 22L scrapie, rPrP-res, or the unsonicated rPrP mixture (No sonic) stained with D13 anti-PrP antibody. A representative region of the thalamus is shown. Mice inoculated with 22L showed clear spongiform change and PrP^Sc^ deposition (brown stain). No spongiform change or PrP^Sc^ was detected in any region of the brain from mice inoculated with rPrP-res. Scale bar = 100 µm.

Immunohistochemical analysis was also used to assay for PrP^Sc^ accumulation in rPrP-res inoculated mice. Sagittal brain sections of C57Bl/10 or CD-1 mice (Figure 4 and Figure S3) inoculated IC with rPrP-res were stained with the anti-PrP antibody D13. In the thalamus (Figure 4 and Figure S3) and all other regions of the brain (data not shown), the level of PrP staining in rPrP-res inoculated mice was identical to that of mice inoculated IC with control mixtures that had not been subjected to PMCA. By contrast, PrP^Sc^ was easily detectable in 22L scrapie-infected C57Bl/10 mice (Figure 4 and Figure S3). Furthermore, standard H&E staining did not detect the spongiosis commonly associated with prion disease (data not shown). Thus, using two different mouse strains and up to three different inoculation routes, we were unable to detect any sign of clinical or subclinical prion infection in mice inoculated with rPrP-res. These data strongly suggest that the rPrP-res generated in our PMCA reactions was not infectious.

### rPrP-res does not Induce the Conversion of PrP^C^ to PrP^Sc^ in Scrapie Susceptible Cell Lines

**Figure 5:**
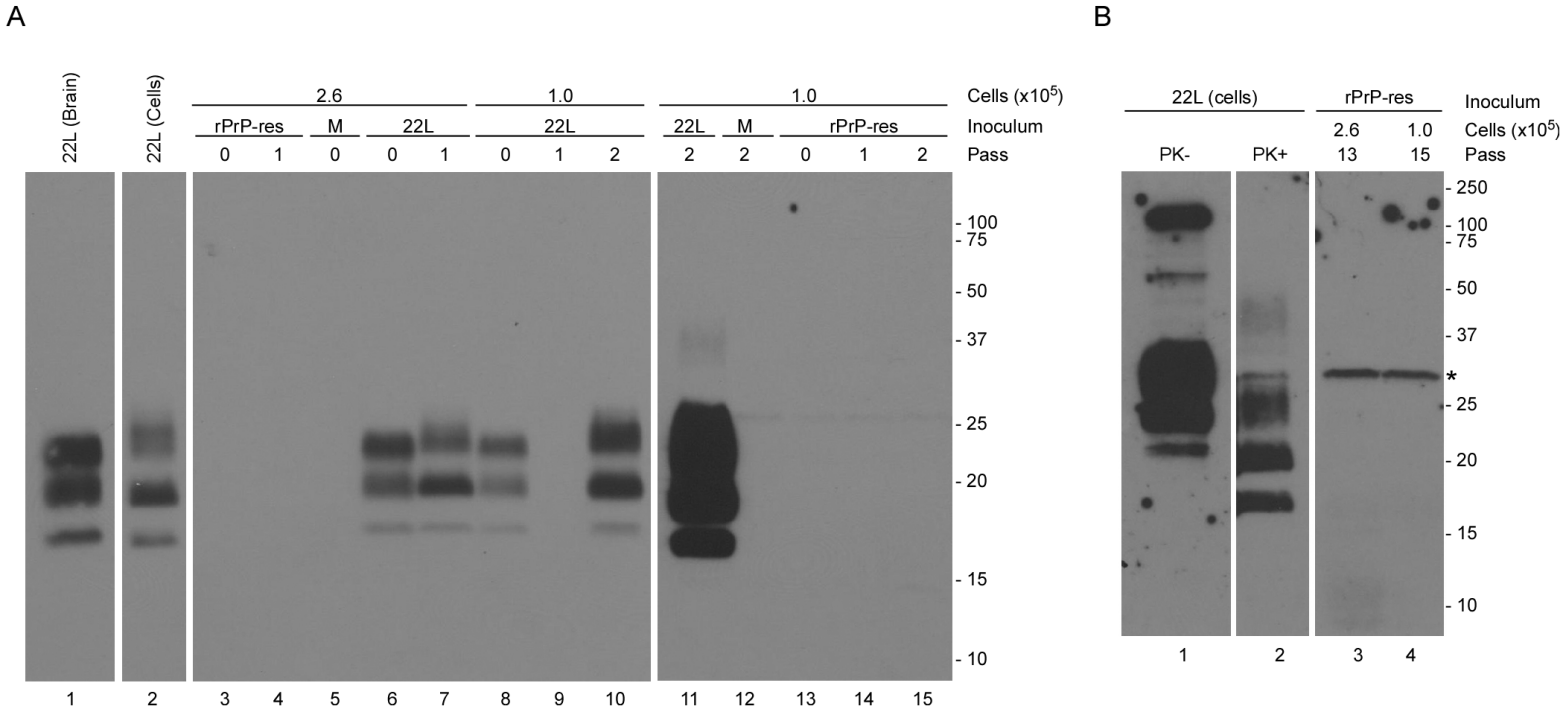
rPrP-res does not induce detectable PrP^Sc^ formation in SN56 cells. SN56 cells plated at a density of 1.0×10^5^ or 2.6×10^5^ cells/ml were inoculated with rPrP-res, brain homogenate from a mouse with clinical 22L scrapie, or cell culture medium alone (M). Inoculated cells were then lysed, digested with PK and analyzed for PrP^Sc^ formation by immunoblot using the anti-PrP mouse monoclonal antibody 6D11. (A) PrP^Sc^ content of cells exposed to brain-derived 22L or rPrP-res prior to passage (0) or after 1 and 2 passages. The first lane shows PrP^Sc^ in a 1% brain homogenate from a 22L-infected mouse and the second lane represents a 1∶100 dilution of PrP^Sc^ derived from SN56 cells persistently infected with 22L. The lack of reactivity in the 22L passage 1 sample is due to loss of the cell pellet during sample processing. Lanes 11–15 represent longer exposures of the same gel shown in lanes 1–10. Thus, lane 11 is just a longer exposure of lane 10 while lanes 12–15 represent the last 4 lanes of the gel. (B) PrP^Sc^ content of cells exposed to rPrP-res at passages 13 and 15. The first lane represents undigested PrP from 22L infected SN56 cells and is 1% of the amount of material loaded in the PK-treated samples. The second lane is a 1∶100 dilution of PrP^Sc^ from SN56 cells persistently infected with 22L. The panel represents a single exposure. Molecular mass markers (kDa) are indicated on the right and the asterisk indicates the location of a cross-reactive proteinase K band. Irrelevant lanes have been removed and some lanes have been rearranged for clarity.

Our data demonstrate that, although rPrP-res could self-propagate in vitro, it was unable to induce PrP^Sc^ formation and clinical disease in mice. This suggested that rPrP-res was unable to convert fully glycosylated and membrane anchored mammalian PrP^C^. Alternatively, it was possible that rPrP-res induced the formation of PrP^Sc^ only transiently. Transient PrP^Sc^ formation, also known as acute PrP^Sc^ formation, has been shown to occur after cells are exposed to prions but prior to passage [34], [44]. Importantly, acute PrP^Sc^ formation can be induced even by strains of mouse scrapie which do not infect susceptible cell lines [34]. In order to determine if rPrP-res could induce the conversion of mammalian PrP^C^ into PrP^Sc^ either acutely or persistently, the scrapie susceptible septal neuron cell line SN56 [31], [45] was plated at different densities and exposed to either 15 ng of rPrP-res, a 1% brain homogenate from mice infected with 22L scrapie, or cell-culture medium alone. After 96 hours (Pass 0) and at selected passages post-infection, lysates from inoculated cell lines were PK digested followed by immunoblotting with the anti-PrP antibody 6D11. No PrP^Sc^ was detected in SN56 cells inoculated with rPrP-res or cell culture medium acutely (Figure 5A, Pass 0), at early passage (Figure 5A, Pass 1 and 2), or at late passage (Figure 5B, Pass 13 and 15). By contrast, SN56 cells inoculated on the same day with 22L scrapie-infected mouse brain homogenate induced PrP^Sc^ formation at passage 0, 1 and 2 (Figure 5A). Thus, the SN56 cells used could support PrP^Sc^ formation and mouse prion infection.

As a further test of the ability of rPrP-res to induce PrP^Sc^ formation, we used a cell line that does not normally express PrP (CF10 cells [32]) which had been stably transfected with a plasmid over-expressing wild-type mouse PrP (CF10+MoPrP cells). These cells are susceptible to scrapie infection [33]. As with the SN56 cell line, inoculation of rPrP-res onto CF10+MoPrP cells resulted in no detectable PrP^Sc^ either acutely (Figure S4A, Pass 0) or persistently (Figure S4B, Pass 6 and 7). These data strongly suggested that the rPrP-res product generated in our PMCA reactions in the presence of lipid and RNA was unable to convert fully glycosylated and membrane anchored PrP^C^ to PrP^Sc^.

### Different Amounts of RNA and POPG do not alter the Ability of rPrP-res to Convert rPrP or PrP^C^

**Figure 6:**
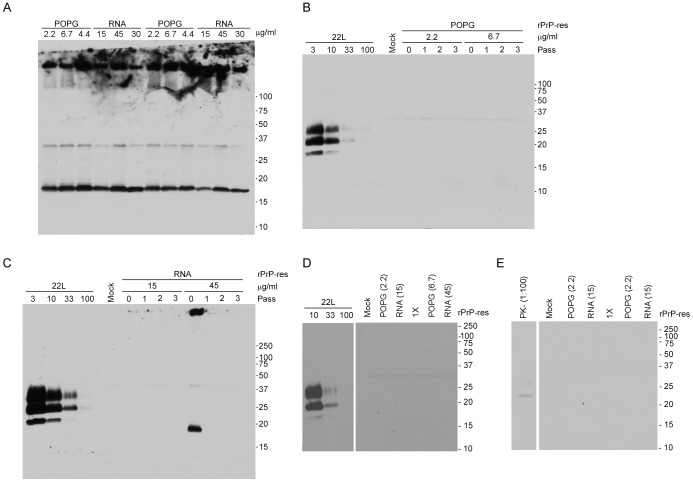
Varying RNA and POPG concentration does not lead to rPrP-res that can induce PrP^Sc^ formation in SN56 cells. (A) rPrP PMCA reactions seeded with rPrP-res and serially propagated in the presence of 30 µg/ml of RNA and 3 different concentrations of POPG lipid (POPG lanes) or in the presence of 4.4 µg/ml of POPG and 3 different concentrations of mouse liver RNA (RNA lanes) were digested with PK and assayed in duplicate by immunoblot. (B) SN56 cells inoculated with cell culture medium alone (Mock) or rPrP-res made in the presence of 30 µg/ml RNA plus 2.2 or 6.7 µg/ml of POPG lipid. (C) SN56 cells inoculated with cell culture medium alone (Mock) or rPrP-res made in the presence of 4.4 µg/ml POPG plus 15 or 45 µg/ml of RNA. (D) SN56 cells inoculated with rPrP-res (Passage 6). The concentration in µg/ml of the RNA and POPG used is indicated in the parentheses. Lanes labeled 1× indicate PMCA products generated using the original POPG and RNA concentrations of 4.4 µg/ml and 30 µg/ml, respectively. (E) SN56 cells inoculated with rPrP-res (Passage 10). Labeling is as in panel D. The first lane is a 1∶100 dilution of undigested PrP from SN56 cells. Based on a standard dilution curve of PrP^Sc^ derived from SN56 cells persistently infected with 22L (first 3–4 lanes in panels B-D), the limit of detection of the immunoblot for PrP is equal to ∼1–3% of the total cell equivalents loaded. For all panels, blots were developed using the anti-PrP mouse monoclonal antibody 6D11. Molecular mass markers (kDa) are indicated on the right of each panel. Irrelevant lanes have been cropped and some lanes have been rearranged for clarity, but each immunoblot panel derives from a single film exposure.

It was possible that generation of rPrP-res capable of inducing the conversion of PrP^C^ to PrP^Sc^ was dependent upon the concentration of POPG and/or RNA in the PMCA reaction. We therefore used rPrP-res to seed PMCA reactions with different ratios of rPrP to lipid or RNA and assayed whether or not the newly made rPrP-res would convert PrP^C^ to PrP^Sc^ in cells. PMCA reactions seeded with rPrP-res generated protease-resistant 16 kDa rPrP-res regardless of the concentration of POPG or RNA used (Figure 6A). Thus, changing the concentration of POPG or RNA in the PMCA reaction did not affect the ability of rPrP-res to self-propagate in vitro. These results are consistent with a recent study demonstrating that complete removal of RNA or POPG does not necessarily prevent propagation of rPrP-res in the presence of a starting seed of rPrP-res [46].

In order to determine if changes in the ratio of rPrP to POPG and RNA would lead to the formation of rPrP-res that could induce PrP^Sc^ formation in cells, the rPrP-res reaction products shown in Figure 6A were concentrated and approximately 15 ng were inoculated in duplicate onto SN56 cells. No cell-derived PrP^Sc^ was detected at any passage following inoculation of the different rPrP-res reaction products (Figure 6B-E), although the rPrP-res inoculum was sometimes visible in inoculated cells lysed without passaging (e.g. Figure 6C, Passage 0). Our results show that even though rPrP-res could be generated in vitro using varying concentrations of lipid and RNA, none of these conditions yielded infectious prions that could stimulate detectable PrP^Sc^ formation in cells.

### The Ultrastructure of rPrP-res is Fibrillar and not Granular

**Figure 7:**
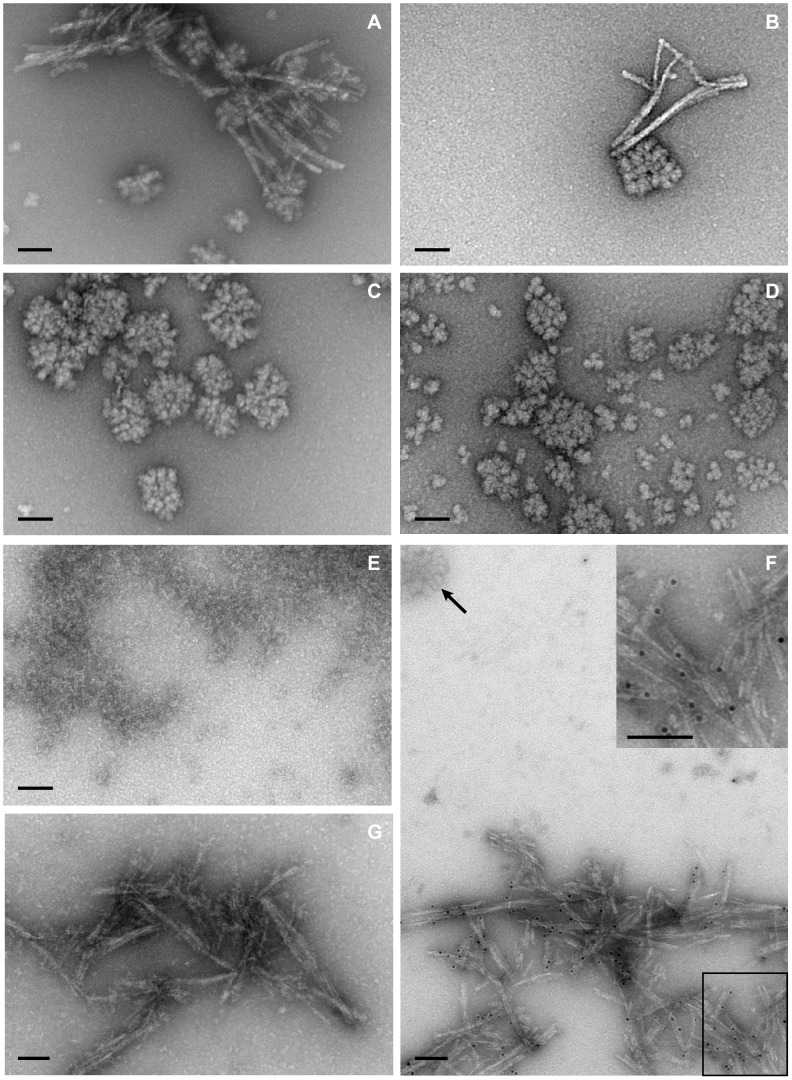
The ultrastructure of rPrP-res is fibrillar and not granular. Transmission electron microscopy by negative stain techniques was used to examine rPrP-res (A, B), unsonicated PMCA substrate (C), mouse liver extract alone (D), and mouse liver extract treated with amylase and amyloglucosidase (E). (F) Fibrils of rPrP-res were immune-labelled with the anti-PrP antibody 6D11 and gold-conjugated anti-mouse IgG secondary antibody, prior to negative staining. The glycogen granule in the upper left of the panel is not immunogenic, while the nearby fibrils are specifically labelled. The inset is magnified 2×. (G) rPrP-res stained with secondary antibody only. Scale bars = 100 nm.

A study by Piro et al. has suggested that infectious prions generated from recombinant PrP in the presence of POPG and RNA are amorphous aggregates and small, spherical aggregates but are not fibrillar [47]. In order to determine if the rPrP-res product generated in our PMCA reactions had a similar ultrastructure, rPrP-res was digested with nuclease and protease, concentrated by ultracentrifugation, and analyzed by transmission electron microscopy. The rPrP-res samples tested contained clusters of fibrils approximately 12 nm wide with a length of up to 500 nm (Figure 7A and B). Similar in width and size to prion fibrils from purified brain-derived PrP^Sc^ [48], [49], these fibrils were not present in unsonicated PMCA reactions (Figure 7C). Immunogold labeling using the anti-PrP antibody 6D11 demonstrated that the fibrils were positive for PrP (Figure 7F) while samples labeled only with the gold-conjugated secondary antibody (Figure 7G) were negative.

The rPrP-res PMCA products generated in this study also contained large numbers of aggregates approximately 25 nm in diameter (Figure 7A and B), similar in size to the 26 nm spherical aggregates previously reported as being associated with infectious rPrP-res [47]. However, these structures were also present in unsonicated PMCA reactions which did not contain rPrP-res (Figure 7C) as well as in mouse liver RNA preparations alone (Figure 7D) indicating that they were not specific to the rPrP PMCA reaction. The ∼25 nm diameter and rosette organization of these structures matches that of glycogen granules, which are known to coprecipitate with RNA during phenol-chloroform extraction [50], [51]. Digestion of the glycogen in our liver RNA samples with amylase and amyloglucosidase removed these granular structures (Figure 7E), confirming that they were glycogen. Furthermore, immunogold staining demonstrated that the glycogen granules were negative for PrP (Figure 7F, arrow). Thus, in contrast to previous work where the structures assigned to infectious rPrP-res were ∼26 nm aggregates [47] or ∼2 nm spheres [46], the only ultrastructural component unique to the non-infectious rPrP-res generated in our PMCA reactions were PrP-positive fibrils.

### Recombinant PrP Purified by Two Different Protocols does not form Infectious rPrP-res

Unlike the previous study of Wang et al. [14], we were unable to generate high levels of infectious rPrP-res in PMCA reactions containing POPG and RNA. However, the recombinant mouse PrP used as a substrate in our study was purified using a slightly different technique. In particular, our purification did not involve the use of BME during PrP refolding, the use of a cation exchange step, or the dialysis of purified PrP into 10 mM sodium phosphate, pH 5.8 (see Materials and Methods). While rPrP produced using our purification method has been shown to generate very low levels of prion infectivity [16], it was possible that its structure was incompatible with conversion to the highly infectious rPrP-res reported previously [14]. We therefore repeated our PMCA reactions using rPrP that had been prepared as closely as possible to the method used in Wang et al. [14] (designated rPrP2) and compared it to PMCA reactions using rPrP prepared according to our original protocol.

**Figure 8:**
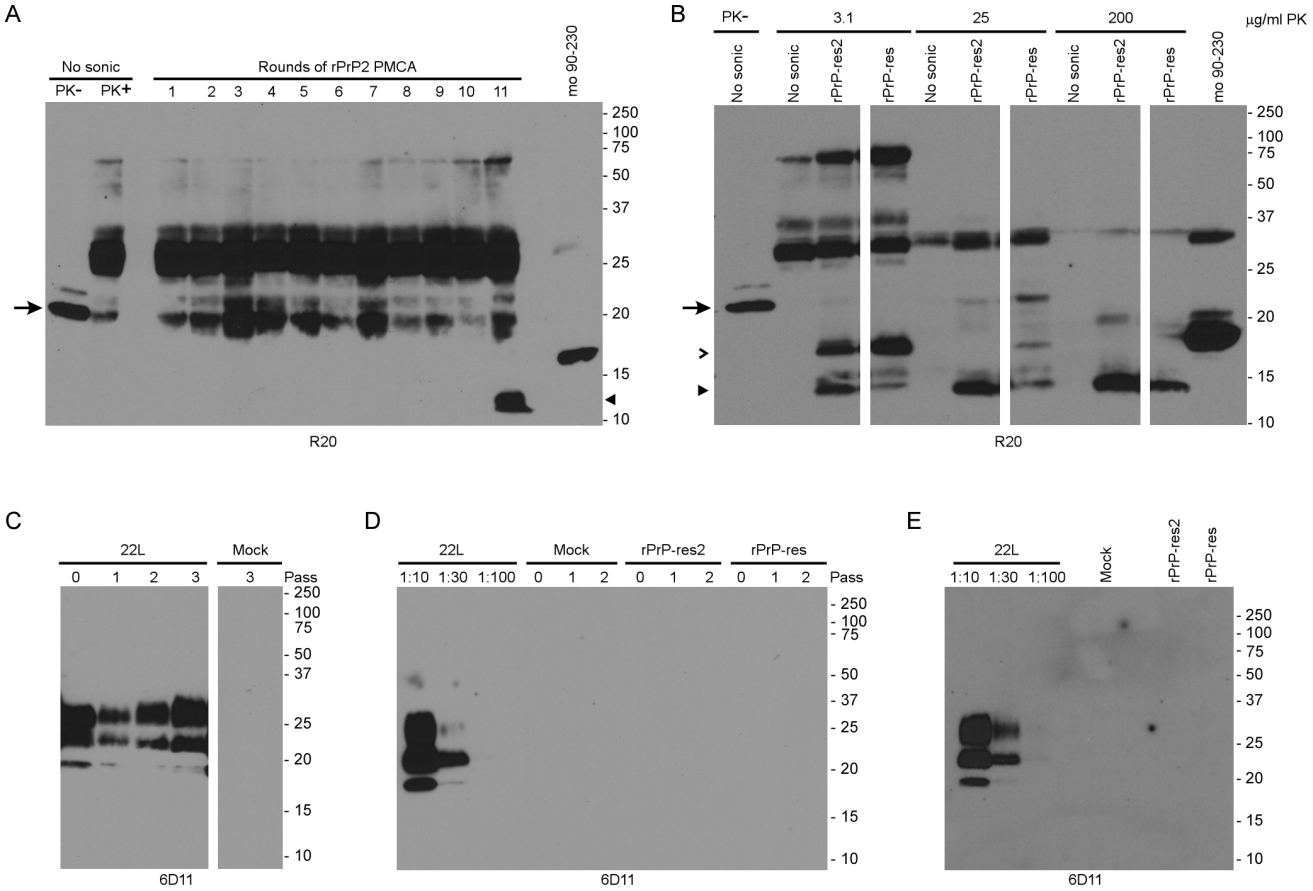
Two different preparations of mouse rPrP do not spontaneously form rPrP-res capable of inducing the formation of mammalian PrP^Sc^ in SN56 cells. (A) The products of eleven rounds of unseeded rPrP2 PMCA were PK digested and assayed by immunoblot with the C-terminal anti-PrP rabbit polyclonal antibody R20. The emergence of a13 kDa rPrP-res2 band (closed arrowhead) can be seen in Round 11. Unsonicated rPrP2 substrate mixture in the absence (PK-) or presence (PK+) of PK is shown in lanes 1 and 2. The PK- control sample was diluted 1∶100 prior to loading. Purified mouse rPrP from residues 90–230 is shown in the last lane as a molecular mass reference. Arrow: rPrP. (B) PMCA reaction products and unsonicated rPrP2 substrate (No Sonic) were digested with eight-fold dilutions of PK and assayed by immunoblot using the C-terminal anti-PrP rabbit polyclonal antibody R20. The PK- control sample was diluted 1∶100 prior to loading (lane 1), while the digested samples were loaded undiluted. Undigested rPrP peptide 23–230 (Lane 1, arrow) and rPrP peptide 90–230 (Lane 11) are used as references for molecular mass. In both panels A and B, the non-PrP bands from ∼18–66 kDa are derived from the digestion products of BSA and PK. Open arrowhead: 16 kDa rPrP-res; Closed arrowhead: 13 kDa rPrP-res. (C-E) SN56 cells were inoculated with cell culture medium alone (Mock), brain homogenate from mice with clinical 22L scrapie (22L), 120 ng of rPrP-res, or 7 ng of rPrP-res2. Cell lysates were PK-digested and analyzed by immunoblot with the anti-PrP mouse monoclonal antibody 6D11 at Passes 0–3 (C), 0–2 (D) and passage 10 (E). All rPrP-res and rPrP-res2 samples were loaded undiluted. In panels D and E, a dilution series of a 22L scrapie-infected cell lysate was used to demonstrate the limit of detection of PrP^Sc^ (Lanes 1–3). For all gels, molecular mass markers (kDa) are indicated on the right. Irrelevant lanes have been cropped but each immunoblot panel derives from a single film exposure.

Of the 24 PMCA reactions containing rPrP2, POPG and RNA which were carried for 20 rounds, two were positive for a protease-resistant product which we designated as rPrP-res2. One of these samples, which was first positive at round 11, is shown in Figure 8A. Consistent with our previous results, 18 of 24 reactions using the rPrP substrate prepared by our original purification method were also positive for a 16 kDa rPrP-res product by round 20 (data not shown). The 16 kDa rPrP-res2 product was PK-resistant and could be truncated to the same 13 kDa protease-resistant core (Figure 8B) described for rPrP-res in our initial experiments (Figure 1). Thus, both rPrP and rPrP2 were capable of generating self-propagating rPrP-res in PMCA reactions containing POPG and RNA.

As a measure of potential prion infectivity, we analyzed the ability of newly generated rPrP-res and rPrP-res2 to induce formation of PrP^Sc^ in cells. The mouse scrapie strain 22L, rPrP-res (120 ng) or rPrP-res2 (7 ng and 90 ng) were inoculated onto SN56 cells as described previously. SN56 cells inoculated with 22L scrapie-infected mouse brain homogenate induced PrP^Sc^ formation at all passes tested (Figure 8C) demonstrating that the cells were susceptible to mouse scrapie infection. By contrast, no PrP^Sc^ was detected in SN56 cells inoculated with cell culture medium (Figure 8C, Mock) or with rPrP-res and 7 ng of rPrP-res2 either acutely (Figure 8D, Pass 0) or at early (Figure 8D) and late (Figure 8E) passage. Similarly, PrP^Sc^ was not detected in SN56 cells inoculated with 90 ng of rPrP-res2 (data not shown). These data are consistent with a lack of prion infectivity in the rPrP-res produced in our PMCA reactions. These results suggest that our inability to produce infectious, recombinant PrP was not solely the result of the method used to prepare the recombinant PrP substrate.

## Discussion

Utilizing a PMCA reaction containing rPrP, synthetic lipid, and mouse liver RNA, we generated rPrP-res with the biophysical hallmarks of disease-associated PrP^Sc^ including the ability to self-propagate, high protease-resistance, and insolubility. However, using multiple routes of infection and two different wild-type mouse strains, we were unable to demonstrate prion infectivity in our rPrP-res PMCA products. In vitro assays for prion infectivity using two different cell lines susceptible to prion infection were similarly negative. Our results demonstrate that the ability to self-propagate into a protease-resistant insoluble conformer is not unique to infectious PrP and that the presence of RNA and lipid cofactors do not necessarily induce PrP to spontaneously refold into a highly infectious form.

At higher concentrations of PK, the 16 kDa rPrP-res protein in our reactions underwent further truncation at the N-terminus leading to a highly protease-resistant 13 kDa rPrP-res protein (Figure 1D). It is possible that the N-terminal truncation of the 16 kDa rPrP-res protein to a 13 kDa fragment is indicative of a conformer of rPrP-res with little or no infectivity. Similarly sized bands have been described when infectious rPrP amyloid fibrils with little or no infectivity are generated in the absence of any cofactors [16], [52], [53] while no 13 kDa species were seen in rPrP PMCA reactions which appeared to generate significant levels of prion infectivity [14]. However, 12–13 kDa protease-resistant PrP isoforms have been identified in different types of human prion disease [54], [55] suggesting that they may play a role in prion pathogenesis. At present, the relevance of these lower molecular weight species to the generation of prion infectivity in vivo or in vitro is unknown.

A second rPrP-res species identified in our PMCA reactions was represented by high molecular weight aggregates that were both PK and SDS-resistant (Fig. 1A). These aggregates did not react with the POM1 and R20 antibodies (Fig. 1C and D) indicating that they were not simply aggregates of the 16 kDa PrP protein but rather a PK-resistant PrP species missing a portion of the C-terminus. They always preceded the appearance of the 16 kDa band by several rounds of amplification and could represent a different species of self-propagating rPrP-res in our PMCA reactions. PrP^Sc^ aggregate size has been correlated with the specific infectivity of prions and larger PrP^Sc^ aggregates tend to have a lower specific infectivity than smaller aggregates [56]. Thus, it is possible that the rPrP-res aggregates in our reactions are too large to trigger PrP^Sc^ formation in vitro or prion infection in vivo. Alternatively, they may interfere and/or compete with the propagation of infectious rPrP-res in vitro.

The rPrP substrate used might also influence the formation of infectious rPrP-res. For this reason, we tested two different rPrP substrates that we prepared according to protocols that generated relatively low [16] or relatively high [14], [26] levels of rPrP-res infectivity. However, even though both preparations were >99% pure and contained ∼ 99% monomeric PrP with an intact disulfide bond (Figure S1C, D and G), neither generated infectious rPrP-res in the PMCA reaction. Some of the substrate appeared to be modified, possibly as a result of oxidation and/or adduct formation (Figure S1E and F). However, these modifications could also be artifacts of the ESI-MS analysis [28], [29]. Oxidation of PrP has been reported to be both beneficial [57]–[59] and detrimental [60] to abnormal PrP formation. We cannot discount the possibility that both of our rPrP substrates contained a population of misfolded or oxidized PrP molecules that either could not refold into an infectious conformation or that inhibited infectious rPrP-res formation. However, no data are available on how modifications to rPrP might influence formation of prion infectivity in vitro. Further study will be needed to understand the potential impact of different recombinant PrP populations on the generation of prion infectivity.

It has been suggested that amyloid forms of rPrP-res are inherently less infectious than non-fibrillar forms [46], [47]. The fact that PrP-positive fibrils were present in our non-infectious rPrP-res PMCA products (Figure 7) but absent in infectious rPrP-res [46], [47] is consistent with this hypothesis. However, it is possible that differences in rPrP-res sample preparation, specifically the use of lower centrifugal force and/or the incomplete removal of the supernatant, may account for the lack of rPrP-res fibrils in previous studies [46], [47]. We were only able to detect rPrP-res fibrils following concentration of our PMCA reaction products via ultracentrifugation and resuspension of the resultant pellet in a small volume (see Materials and Methods). Moreover, it is unlikely that fibrillar structure alone is indicative of less infectious prions. In vivo studies have clearly shown that the presence of PrP fibrils does not necessarily correlate with low levels of prion infectivity. A transgenic mouse model of prion infection where PrP^Sc^ is deposited entirely as amyloid has levels of infectivity greater than or equal to those found in a wild-type mouse model where PrP^Sc^ is not deposited as amyloid [61]–[63].

Small spherical aggregates of ∼26 nm have been reported in preparations of highly infectious rPrP-res and are thought to be representative of the ultrastructure of the infectious particle [47]. We found similarly sized spherical aggregates of ∼26 nm, as well as larger clusters of these aggregates, in our rPrP-res preparations (Figure 7). However, since there was no infectivity associated with the rPrP-res generated in our PMCA reactions (Table 1) and since our spherical aggregates did not contain PrP (Figure 7F), the particles we observed could not be related to prion infectivity. Based on digestion with amylase and amyloglucosidase (Figure 7E), we identified these structures as glycogen granules present as contaminants in the mouse liver RNA used in the PMCA reactions (Figure 7D). The identification of these aggregates as glycogen contaminants derived from the purified RNA used as a cofactor in the PMCA reaction explains the absence of these structures in a recent study using infectious rPrP-res propagated in the absence of RNA [46]. In that study, the predominant ultrastructural components associated with infectious rPrP-res were small, 2 nM spheres [46]. Our data suggest that, unless specifically removed, glycogen granules unrelated to prion infectivity will be present in any PMCA reaction using non-synthetic RNA as a cofactor and may confound the ultrastructural analysis of infectious rPrP-res. Finally, although we did not observe a direct interaction between glycogen and rPrP (Figure 7F), under certain conditions glycogen can associate with rPrP and accelerate its refolding into a structure rich in β-sheet [64]. Thus, its presence could conceivably have an impact on the generation of infectious rPrP-res in vitro.

The de novo generation of prions with apparently high infectivity from bacterially-derived rPrP has only been accomplished in the presence of RNA and lipid cofactors [14]. Our results indicate that the presence of these cofactors does not necessarily restrict the refolding reaction to produce an infectious form of rPrP. Rather, they suggest that the de novo generation of infectious rPrP-res may have a stochastic element capable of yielding multiple conformations, only some of which may be infectious. In the absence of a template of bona fide infectious prions to guide the conversion process, an unseeded PMCA reaction may generate rPrP-res variants which differ unpredictably between reactions. In comparison with PMCA reactions using brain-derived PrP^C^, the lack of N-linked glycosylation and the GPI anchor in rPrP could even exacerbate the generation of a greater repertoire of rPrP-res conformations by removing the structural constraints imposed by these complex modifications.

Competition between different serially propagating rPrP-res conformations would likely favor those that replicate most efficiently in the PMCA reaction. As our study demonstrates, however, this does not necessarily correlate with a conformation capable of triggering disease. This may be because different selective pressures are present in vivo that eventually favor conformations of PrP which are not only able to self-propagate and spread through an organism but are also capable of spread between individuals. Many proteins including alpha-synuclein [65], amyloid β protein [66], [67], and tau [68] all have the prion-like ability to propagate amyloid in vitro and can even spread from cell to cell in some transgenic mouse models. However, they are not associated with the ability to transmit disease between individuals. Our data show that prion protein is also capable of self-propagation in the absence of infectivity and suggest that the ability of protease-resistant prion protein to replicate its own conformation is not necessarily linked to the generation of prion infectivity.

## Supporting Information

Figure S1 **Analysis of recombinant PrP samples by mass spectrometry.** Purified rPrP (left panels) and rPrP2 (right panels) were analyzed by ESI-MS and by silver stain. (A,B) Mass spectra and (C-D) deconvoluted spectra show calculated masses of 23061.85 (rPrP) and 23061.83 (rPrP2). The expected mass of rPrP is 23,063 Da. (E) Deconvolution of ESI-MS spectra for rPrP and rPrP2 (F). Peaks are labeled by molecular mass, with the smaller peaks consistent with modifications that occurred either during purification or the subsequent analysis. The tables list the observed mass and percentage of total area represented by each peak. (G) Silver stain of rPrP (left) and rPrP2 (right) after loading 100 ng of each protein. Consistent with the ESI-MS, SDS-PAGE shows no evidence of higher molecular weight oligomers or lower molecular weight PrP fragments. Samples were run on the same gel and the white space in between represents the deletion of irrelevant lanes. The analyses are consistent with ≥99% sample purity for each of the proteins used in this study. Molecular mass markers are shown on the left.
(TIF)

Figure S2 **CD-1 mice inoculated with rPrP-res do not accumulate detectable PrP^Sc^ up to 367 days post inoculation.** Brain and spleen samples from CD-1 mice inoculated with rPrP-res or the non-PK digested, unsonicated rPrP substrate mixture (No sonic) were homogenized, PK digested, and assayed by immunoblot using the anti-PrP mouse monoclonal antibody 6D11. (A) Brain, 367 dpi. The first lane shows the level of PrP^Sc^ present in a 1∶100 dilution of brain homogenate from a C57Bl/10 mouse inoculated with 22L scrapie. (B) Spleen, 367 dpi. (C) Spleen, 367 dpi, secondary antibody only. Samples assayed are identical to those in panel B. For all panels, tissue samples were loaded undiluted unless otherwise noted. A standard curve of undiluted (UD) or diluted 22L mouse spleen homogenate containing PrP^Sc^ was loaded on the gels in B and C and used to estimate the detection limit of the immunoblot for PrP^Sc^ as detailed in the Materials and Methods. A, B, and C represent individual mice assayed at each time point. Molecular mass markers (kDa) are indicated on the right.
(TIF)

Figure S3 **Lack of spongiform change and PrP^Sc^ in CD-1 mice inoculated with rPrP-res.** Sagittal sections from CD-1 mice inoculated with rPrP-res or the unsonicated rPrP substrate mixture (No sonic) stained with the anti-PrP D13 antibody. A representative region of the thalamus is shown. For comparison, the upper panel is a sagittal section of the thalamus from a C57Bl/10 mouse clinically ill with 22L demonstrating clear spongiform change and PrP^Sc^ deposition (brown stain). No spongiform change or PrP^Sc^ was detected in any region of the brain from mice inoculated with rPrP-res. Scale bar = 100 µm.
(TIF)

Figure S4 **rPrP-res does not induce detectable PrP^Sc^ formation in CF10+MoPrP cells.** CF10+MoPrP cells were inoculated with rPrP-res, brain homogenate from mice with clinical 22L scrapie, or cell culture medium alone (Mock) and analyzed at early (A) and late (B) passages. Cell derived PrP^Sc^ was observed only in 22L inoculated cells. All samples were digested with PK except where noted. Samples that were not treated with PK are equivalent to 1% of the PK-treated samples. Molecular mass markers (kDa) are indicated on the right of each panel. A cross-reacting proteinase K band can be seen can be seen in panel A (asterisk). Irrelevant lanes have been removed and some lanes have been rearranged for clarity, but each immunoblot panel derives from a single film exposure.
(TIF)
