# Supplementary figures and images for: Recombinant Prion Protein Refolded with Lipid and RNA Has the Biochemical Hallmarks of a Prion but Lacks In Vivo Infectivity
Source: PLoS One. 2013 Jul 30;8(7):e71081. doi: 10.1371/journal.pone.0071081 (PMC3728029; doi:10.1371/journal.pone.0071081)

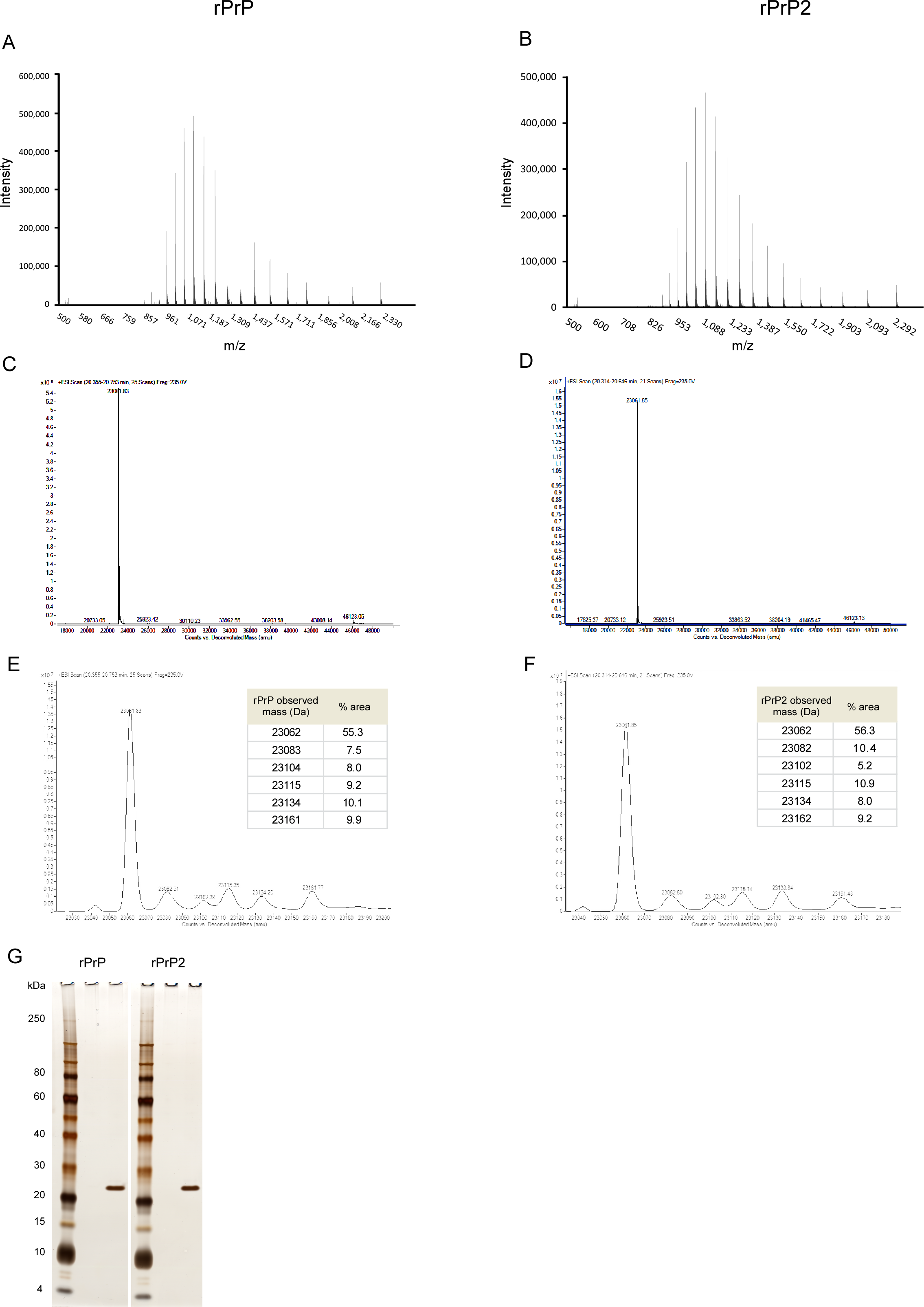

Supplement: Figure S1 — Analysis of recombinant PrP samples by mass spectrometry. Purified rPrP (left panels) and rPrP2 (right panels) were analyzed by ESI-MS and by silver stain. (A,B) Mass spectra and (C-D) deconvoluted spectra show calculated masses of 23061.85 (rPrP) and 23061.83 (rPrP2). The expected mass of rPrP is 23,063 Da. (E) Deconvolution of ESI-MS spectra for rPrP and rPrP2 (F). Peaks are labeled by molecular mass, with the smaller peaks consistent with modifications that occurred either during purification or the subsequent analysis. The tables list the observed mass and percentage of total area represented by each peak. (G) Silver stain of rPrP (left) and rPrP2 (right) after loading 100 ng of each protein. Consistent with the ESI-MS, SDS-PAGE shows no evidence of higher molecular weight oligomers or lower molecular weight PrP fragments. Samples were run on the same gel and the white space in between represents the deletion of irrelevant lanes. The analyses are consistent with ≥99% sample purity for each of the proteins used in this study. Molecular mass markers are shown on the left. (TIF) [file pone.0071081.s001.tif]

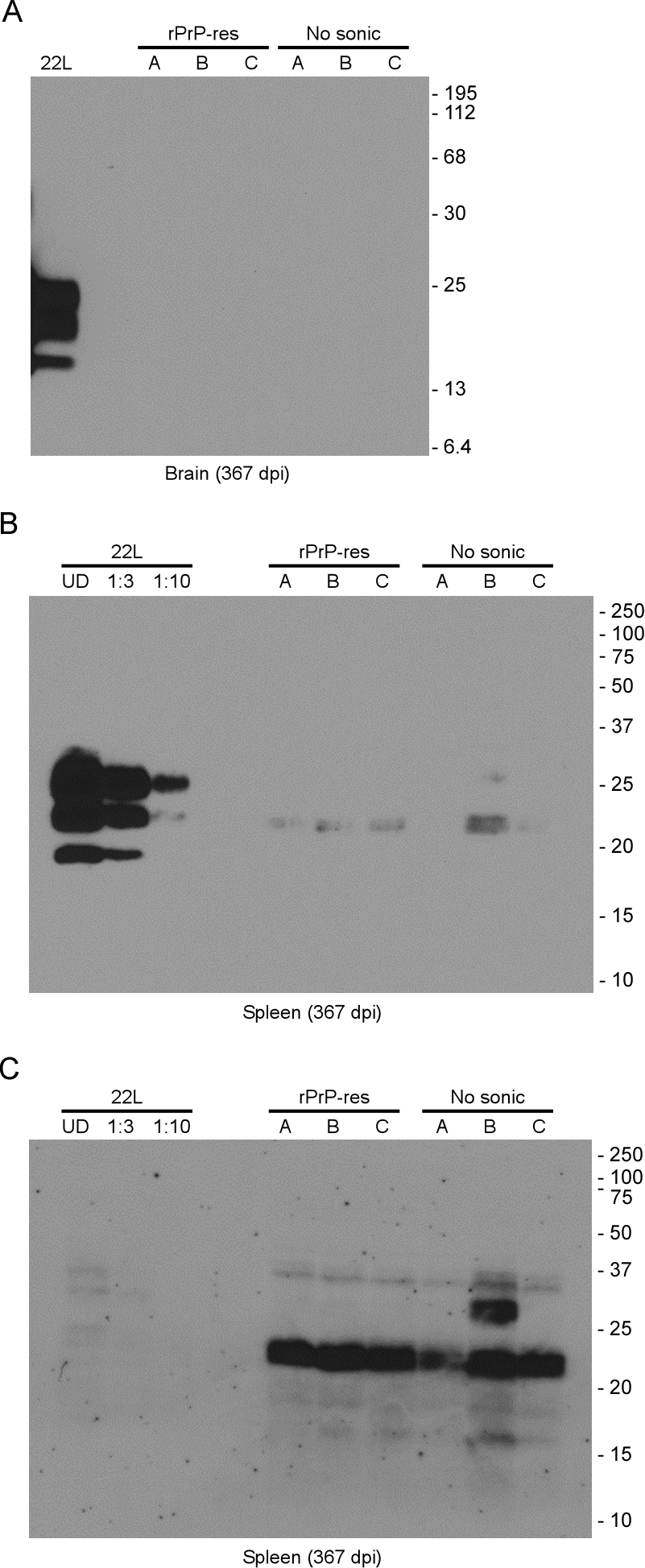

Supplement: Figure S2 — CD-1 mice inoculated with rPrP-res do not accumulate detectable PrPSc up to 367 days post inoculation. Brain and spleen samples from CD-1 mice inoculated with rPrP-res or the non-PK digested, unsonicated rPrP substrate mixture (No sonic) were homogenized, PK digested, and assayed by immunoblot using the anti-PrP mouse monoclonal antibody 6D11. (A) Brain, 367 dpi. The first lane shows the level of PrPSc present in a 1∶100 dilution of brain homogenate from a C57Bl/10 mouse inoculated with 22L scrapie. (B) Spleen, 367 dpi. (C) Spleen, 367 dpi, secondary antibody only. Samples assayed are identical to those in panel B. For all panels, tissue samples were loaded undiluted unless otherwise noted. A standard curve of undiluted (UD) or diluted 22L mouse spleen homogenate containing PrPSc was loaded on the gels in B and C and used to estimate the detection limit of the immunoblot for PrPSc as detailed in the Materials and Methods. A, B, and C represent individual mice assayed at each time point. Molecular mass markers (kDa) are indicated on the right. (TIF) [file pone.0071081.s002.tif]

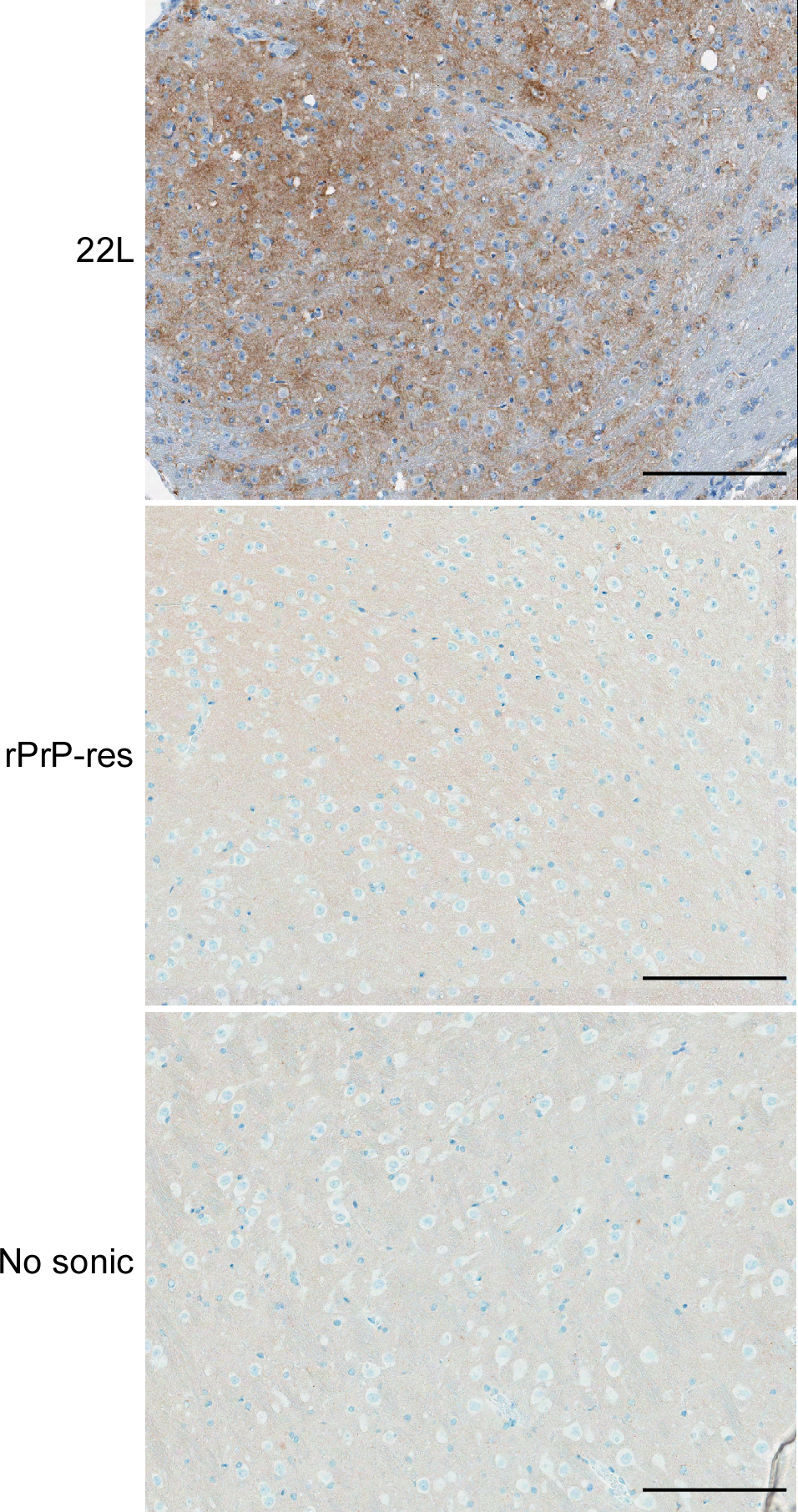

Supplement: Figure S3 — Lack of spongiform change and PrPSc in CD-1 mice inoculated with rPrP-res. Sagittal sections from CD-1 mice inoculated with rPrP-res or the unsonicated rPrP substrate mixture (No sonic) stained with the anti-PrP D13 antibody. A representative region of the thalamus is shown. For comparison, the upper panel is a sagittal section of the thalamus from a C57Bl/10 mouse clinically ill with 22L demonstrating clear spongiform change and PrPSc deposition (brown stain). No spongiform change or PrPSc was detected in any region of the brain from mice inoculated with rPrP-res. Scale bar = 100 µm. (TIF) [file pone.0071081.s003.tif]

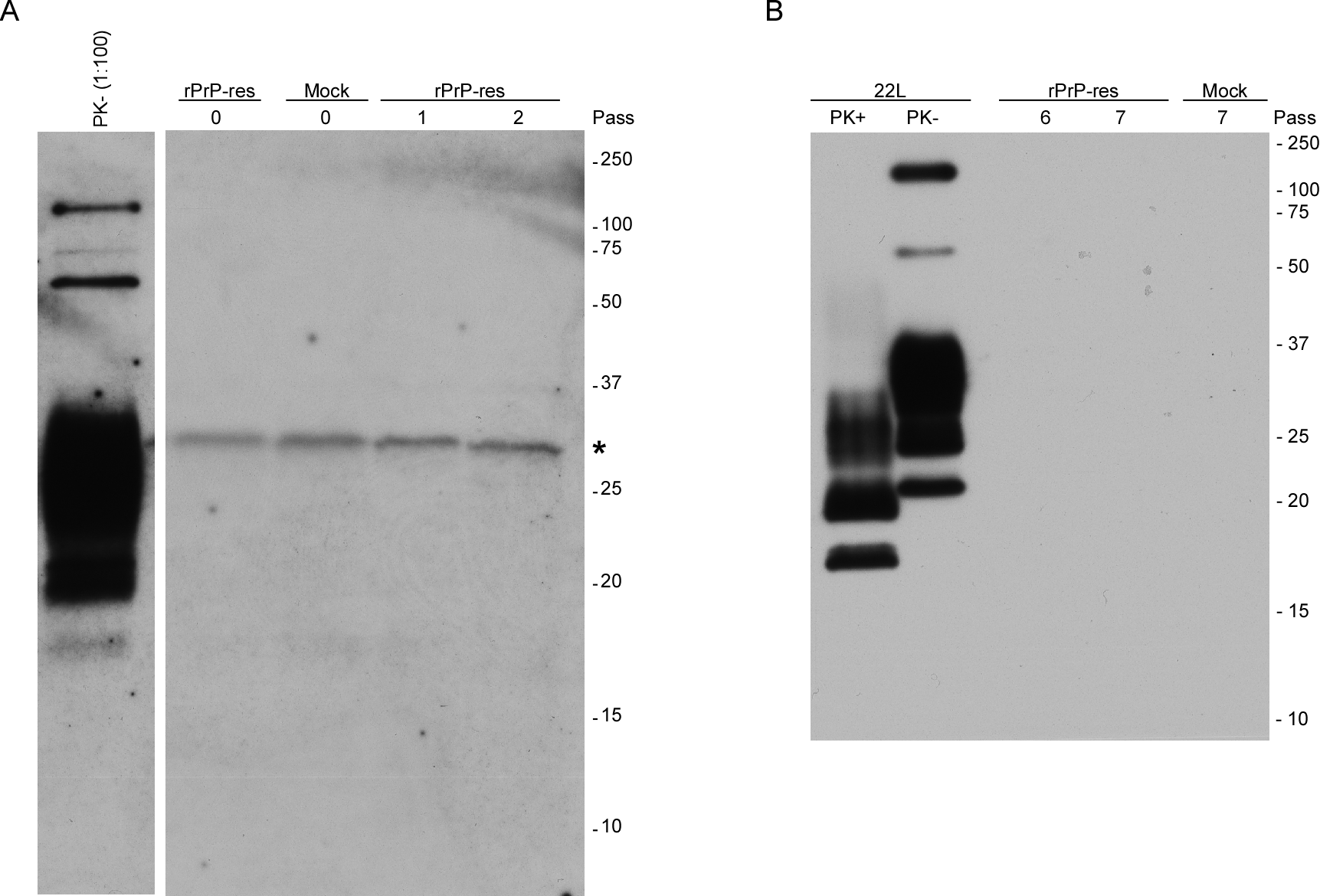

Supplement: Figure S4 — rPrP-res does not induce detectable PrPSc formation in CF10+MoPrP cells. CF10+MoPrP cells were inoculated with rPrP-res, brain homogenate from mice with clinical 22L scrapie, or cell culture medium alone (Mock) and analyzed at early (A) and late (B) passages. Cell derived PrPSc was observed only in 22L inoculated cells. All samples were digested with PK except where noted. Samples that were not treated with PK are equivalent to 1% of the PK-treated samples. Molecular mass markers (kDa) are indicated on the right of each panel. A cross-reacting proteinase K band can be seen can be seen in panel A (asterisk). Irrelevant lanes have been removed and some lanes have been rearranged for clarity, but each immunoblot panel derives from a single film exposure. (TIF) [file pone.0071081.s004.tif]
